# Resolving host–pathogen interactions by dual RNA-seq

**DOI:** 10.1371/journal.ppat.1006033

**Published:** 2017-02-16

**Authors:** Alexander J. Westermann, Lars Barquist, Jörg Vogel

**Affiliations:** 1 RNA Biology Group, Institute for Molecular Infection Biology, University of Würzburg, Würzburg, Germany; 2 Helmholtz Institute for RNA-based Infection Research (HIRI), Würzburg, Germany; Stony Brook University, UNITED STATES

## Abstract

The transcriptome is a powerful proxy for the physiological state of a cell, healthy or diseased. As a result, transcriptome analysis has become a key tool in understanding the molecular changes that accompany bacterial infections of eukaryotic cells. Until recently, such transcriptomic studies have been technically limited to analyzing mRNA expression changes in either the bacterial pathogen or the infected eukaryotic host cell. However, the increasing sensitivity of high-throughput RNA sequencing now enables “dual RNA-seq” studies, simultaneously capturing all classes of coding and noncoding transcripts in both the pathogen and the host. In the five years since the concept of dual RNA-seq was introduced, the technique has been applied to a range of infection models. This has not only led to a better understanding of the physiological changes in pathogen and host during the course of an infection but has also revealed hidden molecular phenotypes of virulence-associated small noncoding RNAs that were not visible in standard infection assays. Here, we use the knowledge gained from these recent studies to suggest experimental and computational guidelines for the design of future dual RNA-seq studies. We conclude this review by discussing prospective applications of the technique.

## Introduction

**Fig 1:**
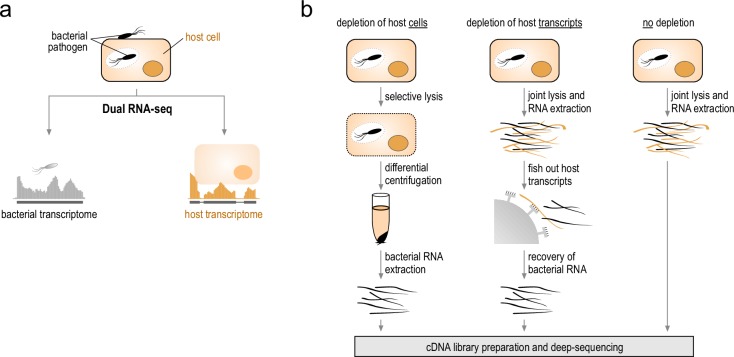
Methods for RNA sequencing of bacterial infections. **A**. Concept of dual RNA-seq. Total RNA is extracted from infected cells and analyzed by RNA-seq. The mixed sequencing reads are assigned to their originating genomes in silico. **B**. Different approaches to quantify gene expression of bacteria in context with mammalian host cells. Traditionally, host material was depleted prior to analysis, either by detergent-mediated lysis of host cells (left) or by sequence-specific removal of host transcripts (middle). Instead, dual RNA-seq omits host depletion (right) and analyzes pathogen and host gene expression in parallel.

The application of high-throughput sequencing–based transcriptomic technologies has delivered major advances in our understanding of biological processes in essentially every organism analyzed [1]. The high resolution of RNA-seq down to the single nucleotide level, however, also allows for a parallel analysis of different organisms interacting with each other—for example, during infection processes (Fig 1A). Simultaneous RNA-seq of host–pathogen models was initiated in the fields of viral [2,3], fungal [4], and parasite infection [5–7], in which the transcriptome structure of the pathogen resembles that of its host. In contrast, bacterial transcriptomes differ dramatically from their eukaryotic counterparts in terms of both the quantity and composition of their RNA (summarized in [8]), which necessitated the use of dedicated protocols to capture bacterial or eukaryotic transcriptomes in isolation. Typically, to profile bacterial gene expression during infection, the overwhelming host material was depleted prior to analysis (Fig 1B). Consequently, until recently transcriptome analyses of bacterial infections were necessarily one-sided, limiting our ability to understand the interactions between pathogen and host.

**Fig 2:**
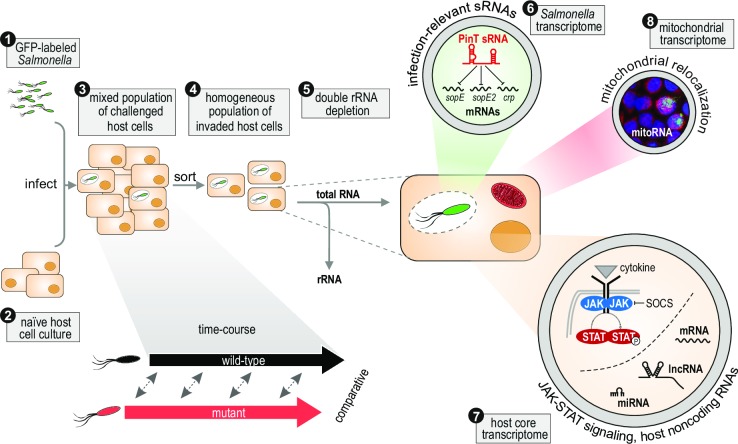
A generic dual RNA-seq workflow analyzing total mixed RNA after double rRNA depletion that discovered the role of PinT small regulatory RNA (sRNA) during *Salmonella* infection of host cells [13]. *Salmonella* having *gfp* stably integrated in their chromosome and expressed from a constitutive promoter were used to infect cultures of HeLa cells. RNA-seq of the bacterial input (1) or mock-infected HeLa cells (2) served as reference controls for *Salmonella* or human gene expression analysis, respectively. Infection was carried out in parallel with wild-type and sRNA mutant *Salmonella* strains, and samples were taken over a time-course of infection. The resulting cell samples constituted a mixed population consisting of both invaded (GFP-positive) and uninfected bystander (GFP-negative) cells (3). To obtain a homogeneous population of invaded cells, the samples were sorted based on the emitted GFP fluorescence (4). Total RNA was extracted from the thus enriched cells, rRNA from both infection partners was depleted (5), and rRNA-free samples were converted into cDNA libraries and sequenced. The resulting sequencing reads were mapped in parallel against the *Salmonella* and human (core and mitochondrial) genome. Differential expression analysis of the time course revealed the strong induction over time of a novel *Salmonella* sRNA, PinT, and comparative analysis unraveled the molecular footprint of this sRNA in the bacterial transcriptome (6). Likewise, comparison of the host transcriptome between wild-type and Δ*pinT* infections revealed PinT-dependent changes in the immune response, including a differential activation of Janus kinase-Signal Transducer and Activator of Transcription (JAK-STAT) signaling as well as changes with respect to the expression of host long noncoding RNAs (lncRNAs) and microRNAs (miRNAs) (7). In addition, the *pinT* status of the infecting bacterium influenced mitochondrial gene expression, and infection with Δ*pinT Salmonella* led to the relocalization of mitochondria in invaded host cells (8).

Five years ago, we coined the term “dual RNA-seq” to refer to the simultaneous RNA-seq analysis of a bacterial pathogen and its infected host (Fig 1A) and theoretically evaluated its feasibility [8]. The key technical issue we identified was the different nature and content of RNA between bacterial and eukaryotic cells. For example, a typical mammalian cell contains on the order of 20 picograms of RNA, which is roughly two orders of magnitude more than a single bacterial cell [9]. Accounting for the prevalence of rRNA transcripts and variable infection rates, this would leave a minute fraction of informative bacterial transcripts in a mixed RNA pool, compromising accurate quantification. This hurdle has now been overcome in a variety of ways (Table 1): by sequencing cDNA libraries to high depth [10], by partially enriching bacterial transcripts prior to sequencing [10,11], by enriching for invaded host cells by fluorescence-activated cell sorting (FACS) [12,13] or laser capture microdissection [14], by depleting rRNA of the bacterium and host either in series or in parallel [10–13,15,16], and by combinations thereof. As a result, most of the current dual RNA-seq protocols [12–15] can provide informative data with as few as ~25 million reads per sample of mixed pathogen–host RNA, making them practical on current sequencing platforms. Importantly, dual RNA-seq of total mixed RNA following double rRNA depletion (see Fig 2) has now become an affordable, straightforward approach that can be generically applied to any bacterial infection model [13].

**Table 1:** Overview of dual RNA-seq and related studies published to date. “Dual SAGE” refers to the simultaneous analysis of host and pathogen by Serial Analysis of Gene Expression (SAGE), and “Multi RNA-seq” refers to a metatranscriptomic analysis of bacterial species constituting the airway microbiota in conjunction with nasal epithelial host cells. “M,” million; “TPM,” transcripts per million; “RPKMO,” reads per kilobase pairs of a gene per million reads aligning to annotated ORFs. Databases containing raw sequencing data: NCBI (National Center for Biotechnology Information), ENA (European Nucleotide Archive), GEO (Gene Expression Omnibus).

	One-sided (focus on bacterial gene expression)					Dual RNA-seq								Dual SAGE	"Multi" RNA-seq
	Mandlik et al. [17]	Lamont et al. [18]	Szafranska et al. [19]	Srikumar et al. [20]	Avican et al. [21]	Humphrys et al. [10]	Vannucci et al. [14]	Mavromatis et al. [11]	Rienksma et al. [15]	Baddal et al. [16]	Avraham et al. [12]	Westermann et al. [13]	Aprianto et al. [22]	Afonso-Grunz et al. [23]	Pérez-Losada et al. [24]
Bacterial species	*Vibrio cholerae*	*Mycobacterium avium subsp*. *paratuberculosis*	*Staphylococcus aureus*	*Salmonella* Typhimurium	*Yersinia pseudotuberculosis*	*Chlamydia trachomatis* serovar E	*Lawsonia intracellularis*	uropathogenic *Escherichia coli* (UPEC)	*Mycobacterium bovis* Bacillus Calmette–Guérin	nontypeable *Haemophilus influenzae*	*Salmonella* Typhimurium	*Salmonella* Typhimurium	*Streptococcus pneumoniae*	*Salmonella* Typhimurium	airway microbiota
Host model	infant rabbits and mice	bovine epithelial cells (MAC-T); primary bovine monocyte–derived macrophages	mouse model of osteomyelitis	murine monocytic cells (RAW 264.7)	FVB/N mice	human epithelial cells (HEp-2)	primary porcine enterocytes	mouse bone marrow–derived macrophages	human monocytic cells (THP-1)	primary normal human bronchial epithelial cells	mouse bone marrow–derived macrophages	diverse cell culture models (human, murine, porcine)	human lung alveolar epithelial cells (A549)	human epithelial cells (HeLa)	nasal epithelium from human donors
Intracellular/extracellular	extracellular	intracellular	extracellular	intracellular	extracellular	obligate intracellular	obligate intracellular	intracellular	intracellular	extracellular	intracellular	intracellular	extracellular	intracellular	extracellular
Sample fixation?	RNA*later*	-	murine tibiae incubated in RNA*later*	RNA stabilization solution (0.2% SDS/19% ethanol/1% acidic phenol)	cryosections incubated in RNA*later*	-	treated with RNase inhibitor prior to embedding	-	-	-	-	RNA*later*	saturated ammonium sulfate solution	-	-
Enrichment of invaded cells?	n.a.	n.a.	n.a.	n.a.	n.a.	-	laser capture microdissection	-	-	n.a.	FACS-based (upon lipopolysaccharide [LPS] staining)	FACS-based (green fluorescent protein [GFP]-expressing bacteria)	n.a.	-	-
Lysis technique	tissue homogenized; cells lysed in TRIzol or lysis/binding buffer (*mir*Vana kit)	TRIzol + zirconium beads	lysostaphin treatment followed by the addition of RLT buffer and mechanic disruption	0.2% SDS/19% ethanol/1% acidic phenol to lyse host cells; TRIzol-based bacterial lysis	Dispomix Drive; glass beads	freeze-thaw + Lysis Solution (MasterPure RNA Purification kit)	Extraction Buffer (PicoPure kit)	Buffer RLT (RN*easy* kit)	TRIzol + bead beating	TRIzol	freeze-thaw	lysis/binding buffer (*mir*Vana kit)	bead beating and phenol-chloroform	lysis/binding buffer (*mir*Vana kit)	TRIzol
RNA extraction technique	TRIzol or *mir*Vana PARIS	TRIzol	RN*easy*	TRIzol	hot phenol	MasterPure RNA Purification	PicoPure	RN*easy*	TRIzol	TRIzol	RNAClean SPRI beads	*mir*Vana	High Pure RNA Isolation	*mir*Vana	TRIzol
Enrichment of bacterial cells/transcripts?	MICROB*Enrich*	MICROB*Enrich* + MassageAmpII	anti-*S*. *aureus* immunoglobulin G (IgG) antibodies coupled to magnetic beads	selective lysis and differential centrifugation	MICROB*Enrich*	with or without polyA-depletion (Poly(A) Purist Mag) to enrich bacterial transcripts; re-combined both RNA samples prior to sequencing	-	MICROB*Enrich*	with or without differential lysis (with guanidine thiocyanate)	-	-	-	-	with or without polyA-enrichment (Dynabeads Oligo dT_25_); poly(A)^+^ and poly(A)^-^ samples analyzed separately	-
rRNA depletion?	MICROB*Express*	-	Terminator Exonuclease	-	MICROB*Express*	RiboZero (gram-negative bacteria; human/mouse/rat)	-	RiboZero (gram-negative bacteria; human/mouse/rat)	RiboZero (epidemiology)	RiboZero (epidemiology)	RiboZero (epidemiology)	RiboZero (epidemiology)	RiboZero (gram-positive bacteria; human/mouse/rat)	RiboZero (gram-negative bacteria; human/mouse/rat)	RiboZero
cDNA library preparation	Illumina: strand-specific ds-cDNA; Helicos: ss-cDNA	mRNA Seq library preparation kit (Illumina)	ScriptSeq	Illumina-based protocol	TruSeq	TruSeq	Ovation RNA-Seq System V2	Digital Gene Expression Tag Profiling kit	TruSeq	ScriptSeq	RNAtag protocol (generation of multiple RNA-seq libraries in a single reaction)	Illumina-based protocol	TruSeq	SuperSAGE libraries for poly(A)^+^ and poly(A)^-^ fractions	TruSeq
Sequencing platform	Illumina (paired-end), Helicos	GA IIx (paired-end)	HiSeq 2500 (single-end)	HiSeq 2000	HiSeq 2000 (paired-end)	HiSeq 2000 (paired-end)	GA IIx (paired-end)	HiSeq 2000 (paired-end)	HiSeq 1500 (paired-end)	HiSeq 2500 (paired-end)	HiSeq 2500	HiSeq; NextSeq 500 (single-end)	NextSeq 500 (single-end)	HiSeq 2000 (single-end)	HiSeq 2500 (single-end)
Sequencing depth/library	~50 M (Illumina); 1–5 M (Helicos)	20 M for bovine samples; 7.5 M for bacterial samples	53–105 M	~20 M	~20–250 M	~14–353 M	~22 M	~15–30 M	~22–40 M (for infection samples)	~60–180 M	on average 6 M	varies (~25 M for main time-course experiment)	on average 70 M	~1–7.5 M	~40 M
Fraction of bacterial reads (of all aligned reads in infection samples)	n.a.	n.a.	0.7%–16.5%	n.a.	0.002%–19%	~0.02% (1 h postinfection); ~30% (24 h postinfection)	~5%	~0.03%–58%	2–4% (nonenriched); 11%–25% (enriched)	~0.2%–1.5%	on average 0.28%	~1%–10%	on average 67%	~0.7% (30 min postinfection); ~2% (24 h postinfection)	~5%
Differential expression analysis tool	DESeq	Cufflinks	edgeR, DESeq, SAMseq	TPM	RPKMO	DESeq	Cuffdiff (Cufflinks)	Cuffdiff (Cufflinks)	edgeR	limma	TPM; DESeq	edgeR	DESeq	TPM	Cufflinks
Data availability	n.a.	PRJNA218473 (NCBI)	PRJEB6003 (ENA)	GSM1462575–1462579, GSM1914919 (GEO)	GSE55292 (GEO)	GSE44253 (GEO)	n.a.	PRJNA256028 (NCBI)	PRJEB6552 (ENA)	GSE63900 (GEO)	GSE65528–31 (GEO)	GSE60144 (GEO)	GSE79595 (GEO)	GSE61730 (GEO)	n.a.

This newfound feasibility has led to a variety of emerging applications of dual RNA-seq to bacterial infection models, including the direct correlation of bacterial gene activity with a specific host response and the identification of “molecular phenotypes” of pathogen genes that are invisible in standard virulence assays [13]. Here, we update our earlier theoretical considerations [8] based on the biological insights gained from recent dual RNA-seq studies of diverse bacterial infection models, aiming to provide experimental and biocomputational guidelines for future dual RNA-seq assays.

## Emerging Applications of Dual RNA-seq

**Fig 3:**
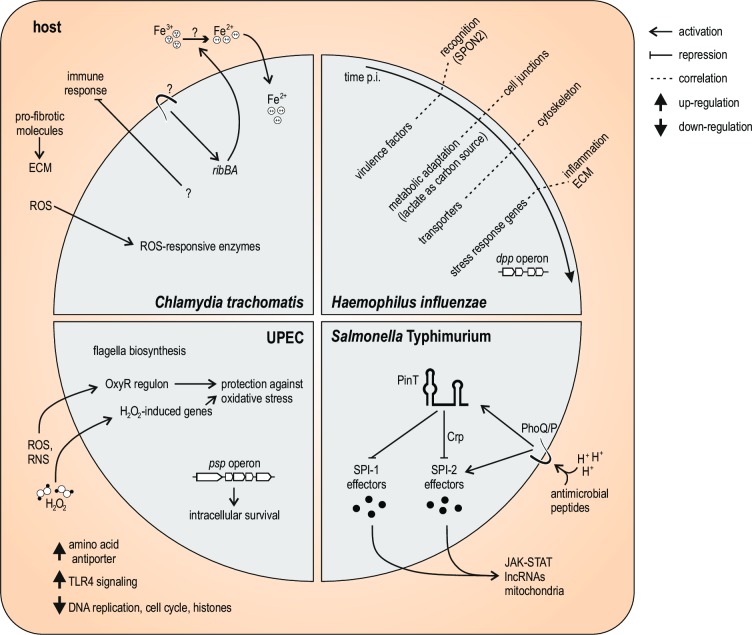
Illustration of biological insights obtained from dual RNA-seq studies in four different bacterial infection models. HEp-2 cells infected with obligate intracellular *Chlamydia trachomatis* [10], primary airway epithelial cells with nontypeable *Haemophilus influenzae* [16], primary murine bone marrow macrophages with uropathogenic *E*. *coli* (UPEC) [11], and diverse human, mouse, and porcine cell lines with *Salmonella* Typhimurium [13]. See main text for details.

Most dual RNA-seq analyses so far have been exploratory, characterizing the transcriptional dynamics of a particular infection system. An early dual RNA-seq study of HEp-2 epithelial carcinoma cells infected with the obligate intracellular pathogen *Chlamydia trachomatis* [10] revealed the induction of numerous metabolic mechanisms early after invasion—for example, riboflavin biosynthesis genes (*ribBA*) responding to extracellular reduction of iron (Fig 3). These changes previously escaped detection because of the few individual *Chlamydia* cells in the infected culture. Host transcripts, on the other hand, revealed an active response to invading *Chlamydia* (albeit with a currently unexplained dampening of immune signaling), in contrast to an earlier microarray-based report in which only few changes in host transcription were observed during early infection [25].

This exploratory concept has been expanded into higher-resolution time-courses covering longer periods of infection. A study following host and pathogen gene expression over 72 hours in primary airway epithelial cells infected with nontypeable *Haemophilus influenzae* [16] revealed a strong early induction in the host of the extracellular pathogen recognition receptor Spondin 2 (SPON2), which acts as an opsonin that promotes macrophage phagocytosis of bacteria in the extracellular matrix [26]. The bacterial transcriptome reflected defined stress responses such as the induction of the *dipeptide transport system permease protein* (*dpp*) operon, whose gene products contribute to the protection against oxidative stress (Fig 3).

A comparative dual RNA-seq approach was taken to study two isolates of uropathogenic *Escherichia coli* (UPEC) strains—one being replication-competent and the other susceptible to killing by the host—in primary murine bone marrow–derived macrophages [11]. While the host transcriptome was broadly similar, bacterial gene expression varied markedly between the two isolates. Several genes were induced exclusively in the replicating isolate, suggesting that some of these might encode for essential virulence factors. Indeed, deletion of one of these genes, *phage shock protein A* (*pspA*), led to a survival defect compared to the cognate wild-type strain (Fig 3).

## Defining “Molecular” Phenotypes by Dual RNA-seq

Our recent dual RNA-seq profiling of *Salmonella* Typhimurium infection of human epithelial cells and porcine macrophages [13] combined these above two strategies of exploratory and hypothesis-driven comparative design (Fig 2). This study, for the first time, also analyzed all major coding and noncoding RNA classes of the bacterial pathogen and its host cell. Within the class of *Salmonella* small noncoding RNAs (sRNAs), the previously uncharacterized *Salmonella* PinT sRNA was consistently and highly induced during infection of 14 distinct cell types. Biocomputational clustering of expression kinetics along a high-resolution time-course of infected HeLa cells predicted that PinT is activated by the PhoP/Q two-component system, which regulates intracellular virulence (Fig 3). Subsequently, a comparative dual RNA-seq time-course with a *pinT* deletion mutant unraveled the function of PinT as a posttranscriptional regulator of the expression of important virulence genes of *Salmonella* inside both human and porcine cell lines. The activity of PinT has widespread effects on the host response, with ~10% of all detected human mRNAs as well as various noncoding transcripts being differentially expressed between the two infections.

Importantly, the generic dual RNA-seq protocol used in this study also detects mitochondrial transcripts, which are typically neglected in host RNA profiling. Analysis of this “third transcriptome” showed that mitochondrial transcripts were hyperexpressed in HeLa cells infected with Δ*pinT* compared to wild-type *Salmonella*. This observation guided the discovery of altered subcellular distributions of mitochondria (Fig 2), an sRNA phenotype that would have likely been missed in standard analyses.

Intriguingly, while PinT does not produce a robust “macroscopic” replication phenotype in cell culture, the dual RNA-seq results show that PinT activity times *Salmonella* virulence gene expression shortly after invasion. We refer to this transcriptional signature as a “molecular phenotype” [27], which may represent a new approach to characterizing the role of gene products in infection. Of note, previous transposon mutagenesis studies in large animal models, including pigs, showed that *pinT* disruption is attenuating [28] despite the absence of an obvious phenotype in cell culture, illustrating the relevance of molecular phenotypes to studying disease in the absence of accessible model systems.

## On Designing a Dual RNA-seq Experiment

While the technical feasibility of dual RNA-seq has now been firmly established, a near-infinite variety of infection models wait to be explored. The complexity of these systems introduces significant challenges for the analysis of the resulting datasets. Next, we will review challenges in planning and analyzing dual RNA-seq experiments.

### (a) Obtaining RNA

Dual RNA-seq requires sufficient starting material for sequencing, particularly for the infecting bacterium. Current protocols are based on at least 10,000 infected cells [12,13,29]. Frequently, only a minor fraction of eukaryotic cells in a sample will be infected, approximately 2%–5% in our study of HeLa cells infected with green fluorescent protein (GFP)-expressing *Salmonella* [13]. Therefore, to enrich for bacterial RNA and to distinguish the host response of infected from noninfected bystander cells, these populations must be separated before analysis. Of the six current dual RNA-seq studies of intracellular bacteria (Table 1), three enriched invaded cells either by laser capture microdissection [14] or via FACS [12,13] using endogenously expressed fluorescent markers and/or cell wall–binding dyes. To minimize unwanted transcriptomic changes during sample acquisition, cells should be kept at low temperature (e.g., sorted under continuous cooling to 4°C [12,29]) until they are lysed. However, when many time points or strains are being compared, it may be challenging to sort the cells immediately upon harvest. In such cases, the transcriptomes should be “frozen” by fixation (Box 1). For example, we have optimized fixation conditions for *Salmonella* infections that leave cells physically intact and do not bleach fluorescent signals or interfere with RNA isolation [13], and recently a similar approach has been used for pneumococcal infections [22].

**Box 1. RNA Preservation**

To minimize unwanted transcriptomic changes during sample processing, the RNA content of infected cells may be stabilized. Two preservation strategies exist: Alcohol- or ammonium sulfate–based preservatives inactivate RNases and RNA polymerases by denaturing cellular proteins through the removal of water. In contrast, formaldehyde-containing fixatives induce intra- and intermolecular cross-links between amino groups and thereby block de novo transcription or RNA decay. In the context of dual RNA-seq, besides leaving cells physically intact to enable cell sorting, transcriptome stabilization must avoid quenching fluorescent signals (as is typically the case for phenol- or alcohol-containing reagents) or interfering with high-quality RNA isolation (which is problematic with cross-linked samples). In our recent study of *Salmonella*-infected HeLa cells, we evaluated eight commonly used transcriptome stabilization techniques (see supplementary material of [13]). For this model system, the ammonium sulfate–based RNA*later* reagent (Qiagen), previously been used to fix infected tissue samples [17,19,21] (Tab. 1) or prokaryotic cells alone [30], performed best. However, this is unlikely to represent a generic protocol. For example, we have seen ex vivo that primary cells, which are more fragile than immortalized cell lines, tend to lyse in RNA*later*. Therefore, transcriptome stabilization should be optimized empirically for any infection model. Promising recent studies have demonstrated the compatibility of paraformaldehyde-based fixation with cell sorting and, importantly, high-quality RNA isolation [31,32]. Detailed discussions of transcriptome fixation are available in the literature [33–35].

Once the infected cells are collected, they must be lysed to extract RNA. Importantly, many standard commercial lysis buffers are optimized only for particular organisms and may, for example, fail to break the thick envelope of gram-positive pathogens. In a study of human THP-1 cells infected with gram-positive *Mycobacterium bovis* [15], total RNA was obtained after mechanically breaking the cells with beads in a benchtop homogenizer. After lysis, a number of RNA isolation methods have been successfully used for dual RNA-seq (Table 1). Before sequencing, it is advisable to first estimate the relative concentration of bacterial and host RNA in the sample (for instance, by quantitative real-time PCR [qRT-PCR] [13]); this can inform decisions about required read depth or whether changes need to be made to the infection protocol to increase bacterial counts, such as increasing the multiplicity of infection.

This naturally raises the question: how many cDNA reads are enough? Differential expression power analyses universally favor biological replication over sequencing depth, particularly once a minimum depth threshold has been attained. For eukaryotes, increasing sequencing depth appears to have diminishing returns after around 10–20 million nonribosomal RNA reads [36,37]—though accurate quantification of low-abundance transcripts may require >80 million reads [38]—while for bacteria this threshold seems to be 3–5 million nonribosomal reads [39]. With current technology, this number of bacterial reads may necessitate specific enrichment, particularly at early time points before intracellular bacteria have undergone replication. However, analysis of subsampled RNA-seq data from a *Vibrio cholera* infection in a juvenal rabbit model [17] showed that differential expression of major virulence and colonization factors could already be detected with as few as 40,000–60,000 nonribosomal RNA reads, in agreement with results for *Salmonella* at early time points of infection [13]. Thus, while low read depth is not ideal, low-coverage data still have value, particularly in the case of poorly characterized pathogens for which basic virulence mechanisms are largely unknown. Clearly, more subtle effects, such as adaptation of bacterial metabolism to the intracellular environment, demand greater sequencing depth.

### (b) Mapping and Normalization

**Fig 4:**
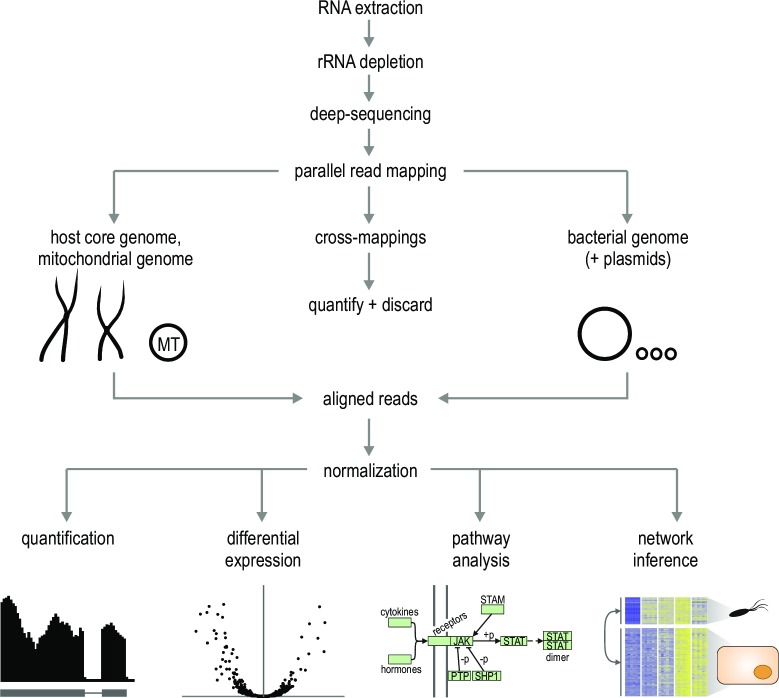
Bioinformatic analysis pipeline for dual RNA-seq datasets. Quality-filtered RNA-seq reads are aligned in parallel against the respective host and pathogen replicons. Reads mapping equally well to both reference organisms (“cross-mappings”) are quantified and discarded from downstream analyses. Reads unequivocally mapped to either the bacterial or host reference are used for quantification and functional analyses. Dual RNA-seq enables a wide range of downstream analyses, discussed in detail in the text. “MT,” mitochondrial genome.

The broad strokes of dual RNA-seq analysis differ little from conventional RNA-seq [40,41]: sequencing reads must be cleaned, mapped, and normalized; differentially expressed transcripts must be identified; and then further functional analyses must be performed to aid in interpretation of the data (Fig 4). However, the complexity of dual RNA-seq designs introduces additional complications at each step as well as entirely new analytical problems.

Much complexity derives from working simultaneously with multiple genome sequences. Although this can be done easily by including all replicons of both organisms as references during mapping, it is important to determine the selectivity of read mapping to both genomes, as cross-mapping reads will affect transcript quantification. In practice, with standard Illumina read lengths of 75–150 bases, we observe negligible cross-mapping in the case of *Salmonella* and mammalian hosts [13], with most of this originating from rRNA and tRNA loci. However, since genome composition varies tremendously across the bacterial phylogeny, potential cross-mapping should be a routine quality control step. The READemption RNA-seq analysis pipeline [42], which relies on the segemehl read mapper [43], contains alignment subcommands implementing such cross-mapping analysis. In principle, any read mapper capable of spliced alignment [44] can be used for read alignment, though some studies have chosen to use separate spliced and nonspliced aligners for mapping to the eukaryotic and bacterial genomes, respectively.

Once mapping has been completed, normalization and quantification are required. Within-sample normalization methods, such as transcripts per million (TPM) [45], often suffice to obtain a qualitative overview of transcriptional dynamics [12,20,46] but should be interpreted with caution since methods are currently lacking for incorporating replicate measurements in these analyses. Most analyses of interest require robust comparisons between samples. The most commonly used (and best performing [47]) RNA-seq normalization techniques address this problem by attempting to scale read counts by a factor derived from a set of putatively invariant genes identified through excluding genes with extreme differences in expression [48], the use of robust median statistics [49], or comparisons of quantiles [50]. These normalization methods make the common assumption that a core set of genes are not differentially expressed and may fail when this assumption is violated. Scenarios violating this assumption have been observed in eukaryotes [51] and can similarly be expected to occur in bacteria after major regulatory transitions, such as that from exponential growth to stationary phase. This may be particularly important in certain infections in which dormant or persister cells develop [52,53]. The use of RNA spikes calibrated to cell counts may enable a robust estimation of differences in expression in such cases [51,54–56]. However, the use of spike-ins presents its own problems: a large multicenter study [57] using External RNA Controls Consortium (ERCC) spike-in controls [58] found that biases introduced in library preparation made absolute transcript quantification unreliable, even when identical protocols and platforms are used. The factors driving these biases are unclear, though they appear to be both sequence- and protocol-dependent [57] and thus may be challenging to correct. This also suggests spike-ins should be added as early as possible in sample processing so that any biases from steps such as ribosomal depletion can be captured. These difficulties notwithstanding, ratios between spike-ins in libraries prepared within the same batch are highly reproducible [57,59], indicating that spike-ins should be sufficient for calibrating most differential expression analyses. New spike-in sets have recently been developed that can be used to assess various aspects of RNA sample processing and analysis [60], such as transcript assembly and isoform quantification, which may be informative in advanced analyses. Alternatively, since dual RNA-seq provides access to two transcriptomes within each pool, if only the host or the bacterium is affected by a global shift in gene expression, a scale factor could be determined for the organism which meets the assumption of the scaling normalization and applied to the other, adjusting for relative population size.

Scaling normalization techniques address differences in sequencing depth between RNA-seq experiments. However, there are many other factors besides read depth which can introduce unwanted variation in high-throughput experiments and lead to reduced power in downstream analyses, commonly referred to as “batch effects” (Box 2). Within the context of dual RNA-seq experiments, myriad opportunities for the introduction of such effects exist: heterogeneity in cell populations and infection, differences in media and reagent batches, variation in laboratory and incubator temperature and oxygen, inaccuracy in cell sorting, and differences in transcriptome stabilization, RNA extraction, library preparation, and sequencing. The prevalence of such effects in high-throughput data has been well documented [61], with lessons to be learned from other fields studying subtle effects in complex model systems, such as stem cell biology and neuroscience [62,63]. We observed similar effects in our study of PinT when comparing wild-type and mutant time-courses [13] and were able to correct for these using recently developed techniques (Box 2).

**Box 2. How to Deal with Batch Effects**

Traditionally, batch effects were accounted for by incorporating date as a nuisance factor in differential expression analysis [64]. While this may work for simple experiments, in complex experiments (such as dual RNA-seq), samples are likely exposed to many treatments that may vary slightly in their effect, and these will not necessarily be constant even within a single “batch.” To solve this problem, recent methods such as RUV-seq and SVA-seq have been developed that perform factor analyses, similar to principal component analysis (PCA), to identify nuisance factors uncorrelated with the experimental factors of interest [54,55]. Nuisance factors can then either be “cleaned” from the read counts directly for the purposes of clustering or other qualitative analyses or incorporated directly as covariates in differential expression analyses. Two excellent case studies provide detailed guidelines for applying methods for evaluating the presence of such confounding batch effects [62,63].

### (c) Differential Expression Analysis

Differential expression analysis forms the backbone of most RNA-seq analyses, most frequently done in the *R* statistical programming language with packages available through the Bioconductor framework [65]. Popular analysis packages include edgeR [66], DESeq2 [67], and limma/voom [68]. These three packages perform well, with slightly different characteristics in benchmarks [69–72]: DESeq generally appears to be more conservative and edgeR more liberal in its *p*-value calculations. While these tools work with predefined annotations and ignore differential isoform usage, RNA-seq also raises the possibility of directly defining boundaries of eukaryotic transcripts, which are typically subject to regulated alternative splicing [73]. A range of algorithms offer isoform discovery, quantification, and differential analysis [74], though generally dedicated pipelines such as the Tuxedo suite [75] have been standard. The recently developed Ballgown utility allows for the easy importation of transcript assemblies and quantifications into *R* [76] and therefore integration of these methods into the Bioconductor RNA-seq analysis ecosystem.

Most of the published dual RNA-seq experiments have involved a time-course and analyzed differential expression by pairwise comparisons; however, this effectively ignores the temporal relationship between samples. Changes in transcript expression can be assumed to be smooth for most genes over time, and this assumption can be used to increase the power of analyses: in effect, contiguous samples act as partial replicates for one another, allowing for more accurate estimation of expression variance. While this does not remove the need for replication, it does raise the possibility of more informative designs than simple replication. For instance, rather than repeatedly sampling the same time points, replicate experiments could be staggered in time so as to provide higher temporal resolution while also demonstrating reproducibility. While not frequently used in the literature, such analyses are possible in analysis packages supporting generalized linear models, such as edgeR and limma/voom, by performing differential expression analysis along fitted curves (see the developmental time-course analysis in *Drosophila* embryos [68]). We hope additional dedicated approaches to time-course analysis will be forthcoming.

### (d) Aiding Interpretation: Functional Analyses

The outcome of differential expression analysis is a long list of genes for both bacteria and host, which must be interpreted in terms of gene function to produce testable hypotheses. Several databases provide suits for this purpose, though none provide complete information for either eukaryotic cells or bacteria. Popular databases include the Gene Ontology (GO) [77] and the Kyoto Encyclopedia of Genes and Genomes (KEGG) [78] databases, which provide general resources for gene functions and interactions in diverse organisms. More specialized knowledge bases also exist—for example, BioCyc [79], which attempts to reconstruct metabolic networks primarily from genomic information. The innate immunity resource InnateDB is of particular interest for the host response part of dual RNA-seq data [80]; it incorporates interaction data from a variety of sources—complemented with manually curated human, murine, and bovine innate immunity pathways and interactions—and provides a number of tools for analyzing and visualizing functional assays in the context of these data. Furthermore, molecular signatures may be reconstructed from relevant high-throughput experiments as collected by MSigDB [81] from eukaryotic microarray and RNA-seq data. The increasing availability of RNA-seq data for bacteria exposed to simple stress conditions raises the possibility that similar signatures could be constructed for them: for instance, by mining resources like the “*Salmonella* Gene Expression Compendium,” which collects expression data for 22 infection-relevant conditions [46].

Dual RNA-seq crucially depends on proper statistical analysis in order to determine gene sets significantly differentially expressed during infection. Originally developed for microarray experiments, many of these techniques remain poorly tested on RNA-seq datasets. Technical issues, such as biases towards detecting differential expression in longer transcripts in sequencing data as compared to array data [82], have made it unclear how applicable these approaches are to RNA-seq. The first benchmarks of gene set enrichment methods on RNA-seq data have recently been published [83] and can provide preliminary guidance.

Dual RNA-seq can also be directly used to infer links between genes through so-called network inference (NI) approaches which are popular in reconstructing global regulatory networks from large collections of expression data in diverse conditions [84,85]. NI methods frequently use measures of coexpression, such as correlation or mutual information, to predict interactions between, for instance, transcriptional regulators and their regulons. With dual RNA-seq time-course gene expression data, a time lag can be introduced in coexpression calculations, allowing for the prediction of potentially causal interactions. This approach was pioneered in studies of the cyanobacterium *Synechocystis* and its response to varying light intensities to identify putative directional interactions [86]. We applied such a correlational analysis to a dual RNA-seq time-course of *Salmonella* infection of HeLa cells, linking virulence gene expression in *Salmonella* to the induction of host immune signaling through the Janus kinase-Signal Transducer and Activator of Transcription (JAK-STAT) pathway [13]. More complex NI models utilizing ordinary differential equations (ODEs) with dual RNA-seq data have successfully predicted host–pathogen interactions between the fungal pathogen *Candida albicans* and murine host cells [4,87]. While ODEs are preferable in that they can explicitly model the dynamics of changes in gene expression and incorporate prior information in a principled fashion, they are also computationally demanding, limiting their use to modeling small subnetworks of genes [88].

## Future Directions

While the potential of dual RNA-seq in cell culture–based infection models has clearly not yet been exhausted, the next steps in the development of this technique are already on the horizon. For example, “Multi RNA-seq” was applied to characterize the human airway epithelium in conjunction with the commensal bacteria populating it [24]; similarly, a recent study profiling *Yersinia pseudotuberculosis* gene expression in the mouse cecum was able to discriminate between various intestinal bacterial species [21] (Table 1). In the future, such approaches could address frequently occurring coinfections of human hosts with bacterial and viral pathogens, including those of *Streptococcus* spp. and influenza virus [89] or *Chlamydia* spp. and human herpes virus [90,91]. As coinfections are a major risk factor for human health [92], such “Triple RNA-seq” experiments would be of direct medical relevance. Likewise, robotic systems have enabled previously prohibitively laborious applications, such as comprehensive chemical–genetic screens [93] and mapping of large transposon mutant libraries [94]. In combination with ongoing improvements in cDNA library preparation and sequencing technologies, these could provide a foundation for high-throughput dual RNA-seq designs. For instance, with efficient multiplexing techniques [29], systematic virulence screens could be imagined that compare expression changes between infections with defined deletion strains of, say, every gene identified as a hit in transposon mutagenesis screens and the isogenic wild-type strain. Combining such ultra-high-throughput approaches will be a powerful strategy to define the molecular phenotypes of hundreds of pathogen genes in parallel, providing a rich basis for dissecting host–microbe interactions [27].

Two more intermediate and exciting possibilities are the expansion to infected tissue (and eventually animal models), and the development of single-cell dual RNA-seq. With respect to the former, several studies suggest widespread differences in bacterial behavior during the infection of two-dimensional monocultures compared to that of three-dimensional tissue [95,96] and whole animal models [97]. Adapting dual RNA-seq to these more realistic models will require numerous innovations. Simple homogenization of the tissue, as has been done for in vivo bacterial RNA-seq studies [19,21] (Table 1), may provide a first step along this path. While this review was in production, two studies were published that report the successful application of dual RNA-seq to in vivo models of infection with extracellular pathogens. Host and pathogen gene expression was analysed in a murine model of acute pneumonia caused by *Pseudomonas aeruginosa* [98] and in a murine gastroenteritis model with *Yersinia pseudotuberculosis* [99]. In both cases, infected tissues (lungs or Peyer’s patches, respectively) were isolated and homogenized prior to total RNA extraction, rRNA depletion, and sequencing. However, since these complex samples do not consist of a single cell type, dissociation of tissues into single-cell suspensions and the enrichment of defined cell types of interest would provide a more complete picture of this complex environment. As dissociation and antibody staining are time consuming, the transcriptomes of host and pathogen must be stabilized immediately after harvest. Ongoing progress in sample preservation provides a foundation on which to build (Box 1) if these treatments can be made compatible with, say, enzymatic treatment to disrupt cell junctions. Additionally, current in vitro dual RNA-seq studies have been performed with 10,000–50,000 sorted cells. Cell numbers will likely be limiting in tissue and animal models, requiring technical advances in cDNA library preparation. Such advances may come in the development of dual RNA-seq protocols for single cells.

Single-cell dual RNA-seq promises to be a game changer in the study of those many bacterial pathogens that are known to form specific, phenotypically distinct subpopulations during infection [100], often associated with distinct disease outcomes [101]. Eukaryotic single-cell RNA-seq studies have already shown that individual immune cells stimulated with the same concentration of the bacterial cell wall component lipopolysaccharide (LPS) mount disparate responses to the challenge [102,103]. In addition, single-cell RNA-seq has revealed heterogeneity in the host mRNA response as the result of pathogen variability [12]. However, current protocols are unable to sample the bacterial transcriptome, as they generally rely on poly(A)-dependent priming of reverse transcription [104,105]. The literature suggests several solutions to poly(A) dependency, such as direct adapter ligation, which unfortunately currently requires approximately10,000 cells [12,13,29]. Priming with random hexamers or—to selectively deplete rRNAs—“not-so-random” primers [106] may provide a more efficient solution. Finally, a thermostable group II intron reverse transcriptase (TGIRT) has recently been described as a highly sensitive (down to 1 ng input RNA), poly(A)-independent enzyme with template-switching activity that can be used to add sequencing adapters, directly avoiding inefficient ligation steps [107,108]. Dedicated bacterial single-cell RNA-seq protocols have also recently been described [109,110] that rely on rolling circle amplification and have been demonstrated to generate large amounts of double-stranded cDNA product from small amounts of input template. Since reverse transcription in these protocols is mediated by random primers, it might be adopted for single-cell dual RNA-seq, though the efficiency of this remains to be tested.

In summary, dual RNA-seq is an emerging technique to profile gene expression changes that accompany infection of mammalian cells by bacterial pathogens. Unlike traditional approaches, dual RNA-seq has proven capable of capturing host and pathogen transcriptomes simultaneously, providing direct insight into host–pathogen interplay. However, dual RNA-seq is still in its infancy, and future efforts—with respect to both experimental aspects and bioinformatics—will be required to exploit its full potential.
